# Rehabilitation Following Above-Knee Amputation in a Pediatric Osteosarcoma Patient: A Case Report

**DOI:** 10.7759/cureus.50859

**Published:** 2023-12-20

**Authors:** Pooja S Ladkhedkar, Aditi Akhuj, Tejaswini Fating, Purva Gulrandhe, Aditi Ambekar

**Affiliations:** 1 Department of Community Health Physiotherapy, Ravi Nair Physiotherapy College, Datta Meghe Institute of Higher Education and Research, Wardha, IND; 2 Department of Community Health Physiotherapy, International Institute of Health Management Research (IIHMR) University, Jaipur, IND

**Keywords:** physiotherapy intervention, quality of life, above-knee amputation, rehabilitation, osteosarcoma

## Abstract

The earliest stages of osteosarcomas are primitive mesenchymal cells. It generally occurs close to the long bones' metaphysis and typically affects the long bones, such as the arm and leg. This case report underscores the pivotal role of physiotherapy in the rehabilitation of a 14-year-old male diagnosed with osteosarcoma, who underwent above-knee amputation. The structured six-week rehabilitation program, encompassing passive, active-assisted, and active exercises for the affected limb, alongside strength training for unaffected joints, produced notable gains in the pain rating scale and the lower-extremity functional scale in just 15 days. These outcomes underscore the significance of early and targeted physiotherapy interventions in optimizing functional outcomes and quality of life for young patients with osteosarcoma after surgery.

## Introduction

The presence of malignant mesenchymal cells that form osteoid is what defines osteosarcoma. They hardly arise from soft tissue; instead, they originate in bone. If left untreated, the illness progresses locally and frequently spreads to other areas. The successful treatment of children with osteosarcoma requires efficient communication among a proficient multidisciplinary team comprising pediatric or medical oncologists, surgeons, pathologists, radiologists, and physiotherapists [1]. In the general population, osteosarcoma affects 2-3 million people a year; however, throughout adolescence, this incidence rises to 8-11 million people annually, peaking between the ages of 10 to 19. Males have always been thought to have a higher incidence of osteosarcoma than females. While osteosarcoma can occur in any bone, it most commonly affects long bones during their metaphysis. With the proximal tibia, proximal humerus, and distal femur being the most often affected around half of the primary sites start at the knee [2]. The first peak occurs during the pubertal growth spurt, which occurs in the age range of 10 to 14 years old. This indicates a strong connection between osteosarcoma and the teenage growth spurt [3,4].

Osteosarcoma most commonly manifests clinically as a painful bone tumor. Knee pain was more noticeable while bearing weight and might apply to the hips or the back. Localized pain, followed by swelling and restricted joint movement, are typical signs of osteosarcoma. Over one-third of the patients had a palpable mass at the initial visit, the most significant clinical finding [5]. Osteosarcoma is now treated with combinations of medications, mostly as neoadjuvant and adjuvant drugs like methotrexate, doxorubicin, and cisplatin; other chemotherapy regimens are also being researched. There is little experience with radiotherapy since osteosarcoma has long been thought to be resistant to radiation dosages that can be used. Since the 1970s, when effective neoadjuvant chemotherapy became available, amputation has been considered a possible osteosarcoma treatment. Still, some surgeons believe that amputation is a preferable alternative for patients with osteosarcoma who have pathologic fractures since prompt and vigorous tumor removal will stop the illness from getting worse due to fractures [6,7].

As a result, during the pre-operative, early post-operative, and rehabilitation periods, measuring levels of gait, balance, and physical activity can provide medical personnel with important information regarding the kind and severity of physical limitations. To improve recovery, this can assist in identifying "at risk" individuals who can benefit from focused treatments and early referrals to rehabilitation [8]. The fear of falling is the anxiety that a person has while they are involved in specific activities. Limiting physical exercise puts an anxious person at a higher risk of cardiovascular illness, diminished physical fitness, and muscular atrophy. Previous research has shown that decreased physical activity has a negative effect on people's quality of life (QOL), fatigue, spasticity, and depression [9]. Future patient-centered therapies and physical activity programs for individuals with lower limb amputations should include the removal of the significant obstacle of fear of falling and devise strategies to mitigate it via the establishment of reasonable yet achievable objectives [10]. For management, psychological support and pre-amputation counseling are important. Treatments for limb fitting and rehabilitation are crucial for optimizing outcomes following amputation. Services should support recovery through early walking assistance and efficient prosthesis repair and maintenance. Both acute and chronic phases require a combination of psychological, occupational therapy, and physiotherapy assistance, as well as access to long-term rehabilitative treatment [11,12]. The rehabilitation of the patient who had surgery for an above-knee amputation is the primary focus of this case study. After following a 15-day rehabilitation plan, improvements were seen in the lower extremity functional scale as well as in the numerical pain rating scale.

## Case presentation

Patient information

A 14-year-old male with right-hand dominance presented with a chief complaint of persistent swelling in his right leg over the past five months, which was gradually progressive and accompanied by significant pain, leading to difficulty in carrying out activities of daily living and compromised mobility. The patient initially sought medical attention at a private healthcare facility, where analgesics were administered to alleviate pain. Subsequently, he was referred to the orthopedic department for further evaluation of persistent swelling and pain, which was primarily localized around the right knee joint. Still, the patient only got symptomatic relief despite medical intervention. Further diagnostic measures, including an X-ray examination and comprehensive blood test, were undertaken. The X-ray revealed the presence of a high-grade conventional osteosarcoma within the right knee joint. Comprehensive blood tests, including complete blood count, kidney function test, liver function test, vitamin D, and calcium levels, demonstrated values within normal ranges.

Clinical findings

**Figure 1:**
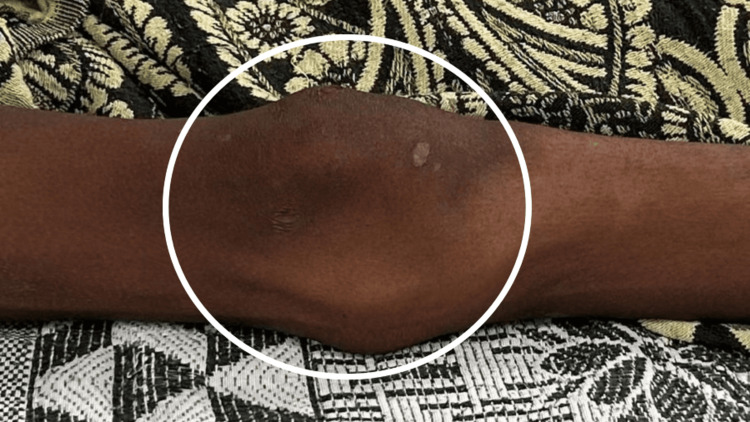
Knee joint The circle shows swelling around the knee joint

Before the commencement of the examination, informed consent was taken from the patient, ethical clearance was done, and he was examined. On observation, the patient was ectomorphic and was seen in a supine lying posture with head end elevated to 30 degrees. The patient was afebrile and hemodynamically stable. Notably, there was evident swelling noted on the right knee. Movement restrictions were observed within the affected joint, and a palpable mass was identified. Prior to the operation, the active range of motion (ROM) was assessed, revealing a range of 20-50 degrees on the right knee and 20-55 degrees on the left knee. Following the surgical intervention, an examination of the operated site revealed the presence of swelling (Figure 1). The incision measured approximately 12 cm in length. Upon palpation, the right knee exhibited warmth and tenderness, graded at level 2. The patient used a visual analog scale to self-report their level of pain, indicating a score of 6/10 during activity and 3/10 at rest prior to the operation. Tables 1, 2 show the ROM of the lower limb and manual muscle testing (MMT), respectively, before the operation.

**Table 1:** Pre-operative ROM of lower limb ROM: range of motion

Parameter	Right lower limb (in degrees)	Left lower Limb (in degrees)
Hip flexion	0-20	0-40
Hip extension	0-15	0-30
Abduction	0-36	0-44
Adduction	0-22	0-40
Knee flexion	0-15	0-30
Knee extension	0-30	0-40

**Table 2:** Pre-operative MMT score 0: No contraction; 1: Flickering contraction; 2: Full ROM with gravity eliminated; 3: Full ROM against gravity; 4: Full ROM against gravity, moderate resistance; 5: Full ROM against gravity, maximal resistance ROM: Range of motion; MMT: Manual muscle testing

Muscles	Right lower limb	Left lower limb
Hip flexors	2/5	3/5
Hip extensors	2/5	4/5
Hip abductors	3/5	3/5
Hip adductors	3/5	4/5
Knee flexors	2/5	3/5
Knee extensors	3/5	3/5

Diagnostic assessment 

**Figure 2:**
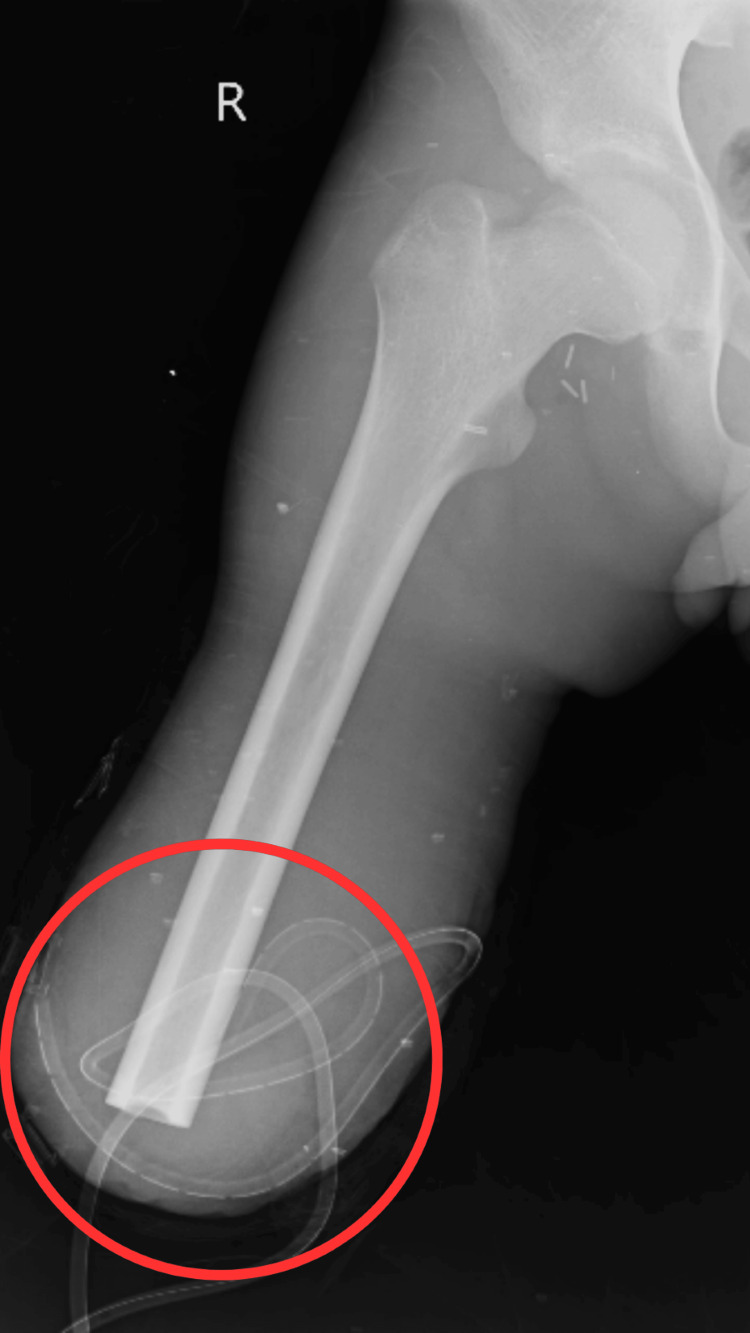
X-ray anteroposterior view The red circle represents the right amputated lower limb

To inspect the condition and amputation, radiographic scans were carried out after the surgical amputation. Figure 2 shows an operative X-ray with the above-knee amputation. 

Surgical procedure

**Figure 3:**
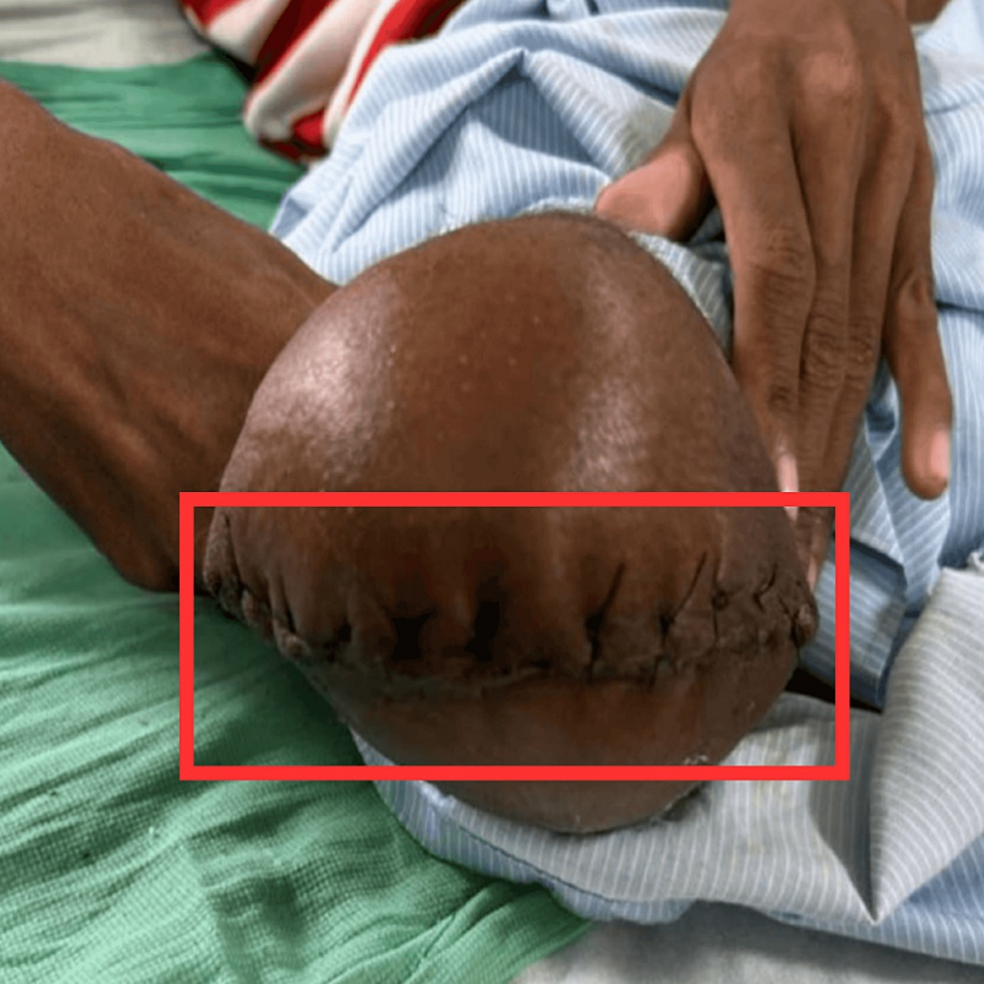
Amputated limb The rectangle shows an incision done about 12 cm circumferentially

The above knee amputation with right modified inguinal lymph node dissection was done under all aseptic precautions, cleaning, painting, and draping of the right knee, and the patient was taken supine on the operation theatre table. Spinal and epidural anesthesia was given, and a circumferential fish mouth incision was taken 10 cm above the knee (Figure 3); soft tissues were dissected in all the planes. Utilizing a bone saw, the femur was transected in a posterior-to-anterior direction, and the distal segment was amputated. The stump was created by myodesis of the hamstring, adductor, and quadriceps group of muscles, and a drain was placed in the intra-muscular layer. Aseptic dressing was performed, and a crepe bandage was applied under compression.

Physiotherapy intervention

Along with the above protocol, the patient was also given some rehabilitation for other joints that are unaffected by strength training.

Phase 1 (0-2 Weeks)

During this initial phase, the patient was provided with comprehensive information about their condition, emphasizing the benefits and significance of physiotherapy. The treatment approach aimed to enhance overall health, mitigate potential complications, and facilitate the patient's ability to perform essential activities such as walking with crutches and ascending stairs.

Passive exercises were administered to the affected leg, with a frequency of three times a day, consisting of 10 repetitions per set. Concurrently, strength training for the unaffected leg was initiated and also conducted three times a day, with 10 repetitions per set. Static quadriceps and static hamstring exercises were performed, involving 10 repetitions with a five-second hold. Additionally, exercises focusing on hip abduction and adduction, with a five-second hold, were introduced, comprising one set of 10 repetitions. Static exercises targeting the gluteal muscles and abdominal muscles, each held for 10 seconds, were prescribed for one set of 10 repetitions. Strengthening exercises for both upper limbs were incorporated, consisting of one set of 10 repetitions. Diaphragmatic breathing exercises were performed for 10 repetitions in one set twice a day. 

Phase 2 (2-4 Weeks)

In this phase, active assisted exercises were introduced for the affected leg, scheduled three times a day, with one set comprising 10 repetitions. Additionally, calf strengthening exercises with a one-kilogram weight were initiated and performed twice daily with 10 repetitions per session. Dynamic quadriceps and dynamic hamstring exercises were introduced, incorporating a five-second hold, and conducted for 10 repetitions per set. Furthermore, resistance-based strengthening exercises for both upper limbs were administered, involving 10 repetitions per set. Bed mobility was taught, and training for getting in and out of bed, toilet, and wheelchair were given.

Phase 3 (4-6 Weeks)

**Figure 4:**
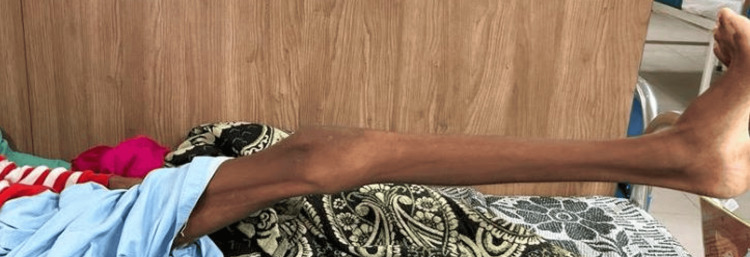
Straight leg raising

**Figure 5:**
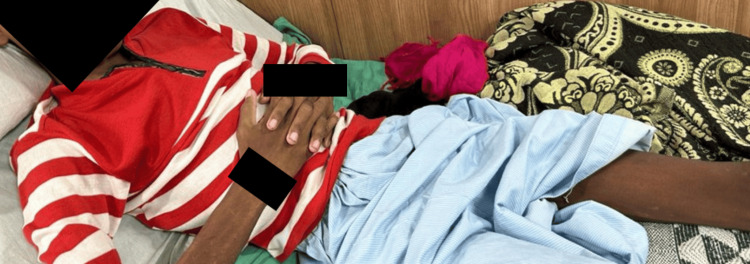
Diaphragmatic breathing exercise

During the third phase, the patient engaged in active exercises for the affected leg, carried out three times daily, with one set comprising 10 repetitions. Calf strengthening exercises, now utilizing a two-kilogram weight, were initiated and performed twice a day, with 10 repetitions per session. Dynamic quadriceps and dynamic hamstring exercises, including a five-second hold, were continued at 10 repetitions per set. Moreover, upper limb strengthening exercises with maximal resistance were prescribed, involving 10 repetitions per set. The patient commenced ambulation with the assistance of a walker. Figures 4, 5 show the patient receiving treatment.

Follow-up and outcome measures

A well-planned physical therapy intervention process was initiated. Every week for six weeks, there was a follow-up, and after discharge, outcome measures were discovered. Tables 3, 4 show the ROM and MMT of the right lower limb. Table 5 shows the outcome measures and scores taken on the first day and the day after the therapeutic intervention.

**Table 3:** ROM of the right lower limb ROM: Range of motion

Parameter	Pre-rehabilitation (day 1) (in degrees)	Post-rehabilitation (day 44) (in degrees)
Hip flexion	0-20	0-65
Hip extension	0-15	0-50
Abduction	0-36	0-44
Adduction	0-22	0-40

**Table 4:** MMT score of right lower limb 0: No contraction; 1: Flickering contraction; 2: Full ROM with gravity eliminated; 3: Full ROM against gravity; 4: Full ROM against gravity, moderate resistance; 5: Full ROM against gravity, maximal resistance MMT: Manual muscle testing; ROM: Range of motion

Muscles	Pre-rehabilitation (day 1)	Post-rehabilitation (day 44)
Hip flexors	2/5	3/5
Hip extensors	2/5	4/5
Hip abductors	3/5	4/5
Hip adductors	3/5	4/5

**Table 5:** Outcome measures

Scales	Pre-rehabilitation	Post-rehabilitation
Numerical pain rating scale	7/10	2/10
Lower extremity functional scale	22/100	55/100

## Discussion

In both children and adults, the most common nonhematologic bone cancer is osteosarcoma. Usually, osteosarcoma develops along the long bones' growing plate [13]. In order to maximize outcomes following amputation, rehabilitation, and limb fitting treatments are essential. Complications with a ROM, including flexion contracture or a decrease in the ROM of the joint, can occur in patients and impact their gait pattern. For such, rehabilitation plays an important role. Predicting the results in recovery after amputation treatments has been found to depend on variations in treatment methods, outcome assessment, and rehabilitative care [14]. Patients with osteosarcoma may benefit from exercise by having a better function, less impairment, and the capacity to maintain their independence and QOL. Exercise can additionally impact the effectiveness of cancer treatments.

Physical exercise has been shown to be beneficial for both pre-operative and post-operative therapy and rehabilitation. Here, we observed the application of a variety of therapeutic strategies, including passive movements, isometric exercises, active assistive movements, and ambulatory techniques, which improved muscle strength and allowed for the achievement of a functional hip range within 15 days of the commencement of physiotherapy rehabilitation [15]. Fiedler et al. illustrated that mobility training must include exercises that improve the amputee limb's voluntary control as well as walking and standing stability. Stressing the value of symmetrical motion patterns, rehabilitative exercises can help lessen these negative impacts [16].

Exercises targeting the hip muscles, namely the hip abductor and hip extensor groups for pelvic stability, should be a part of the lower-extremity program. In hamstrings and quadriceps muscles, when a prosthetic device is utilized, knee stability is essential, and the strength of the transtibial residual limb is critical for this. It's possible to convert conventional open-chain exercises into closed-chain workouts that are more focused on the way muscles work when walking [17]. To raise the performance level as soon as possible, it is imperative to reduce the bilateral deficit and the level of atrophy. When the muscles of a healthy limb are trained, the muscles of the amputated limb should react noticeably and favorably, which may be anticipated by selecting an appropriate strength and endurance program [18]. Individuals who have lost a lower limb appear to gain physical advantages from lifestyle therapies that emphasize physical exercise and stress reduction. Regular physical exercise and sports participation improve psychological health, self-esteem, and coping mechanisms before and after the loss of limb [19]. Bouzas et al. explain that in adults who wear prosthetics and have had one lower limb amputated, the combination of functional physical activity and muscular endurance exercise appears to have stronger favorable impacts on levels of functionality, muscular fitness, and cardiorespiratory fitness [20].

## Conclusions

A malignant mesenchymal cell tumor named osteosarcoma may affect the age group between 10 and 14 years. It mostly affects the body's long bones. Pain, swelling, and limited ROM in the affected joints are some of its symptoms. It may get severe if left untreated. Amputation is a surgical procedure for treating osteosarcoma. Physiotherapy plays a vital role in the rehabilitation process after the surgery and brings back the individual to normal daily life. This physiotherapy regimen contributed to the restoration of muscle strength in the amputated limb and facilitated the refinement of gait patterns through the utilization of walking aids such as a walker or crutches. Early implementation of physiotherapeutic rehabilitation emerges as a pivotal factor in averting post-amputation complications, with strengthening and retraining protocol leading to the enhancement of QOL and augmented functional independence.
